# 2375. Contemporary Patterns and Predictors of Influenza and COVID-19 Vaccination Among US Adults During the COVID-19 Pandemic

**DOI:** 10.1093/ofid/ofad500.1996

**Published:** 2023-11-27

**Authors:** Christopher Bush, Katherine E Mues, Shaina Desai, Brent Arakaki, Priyadarshani Dharia, Parinaz Ghaswalla, Yoonyoung Park

**Affiliations:** Aetion, Inc., New York, City, New York; Aetion, Inc., New York, City, New York; Aetion, Inc., New York, City, New York; Aetion, Inc., New York, City, New York; Moderna, Inc., Cambridge, Massachusetts; Moderna, Inc., Cambridge, Massachusetts; Moderna, Inc, Belmont, Massachusetts

## Abstract

**Background:**

With the development of combined influenza and COVID-19 vaccines, there is a need to understand contemporary patterns of both COVID-19 and influenza vaccine administration in the real world. This study aimed to describe patterns and assess predictors of COVID-19 and influenza vaccine administration among insured, influenza-vaccinated adults in the US from 2020 to 2022.

**Figure 1.**
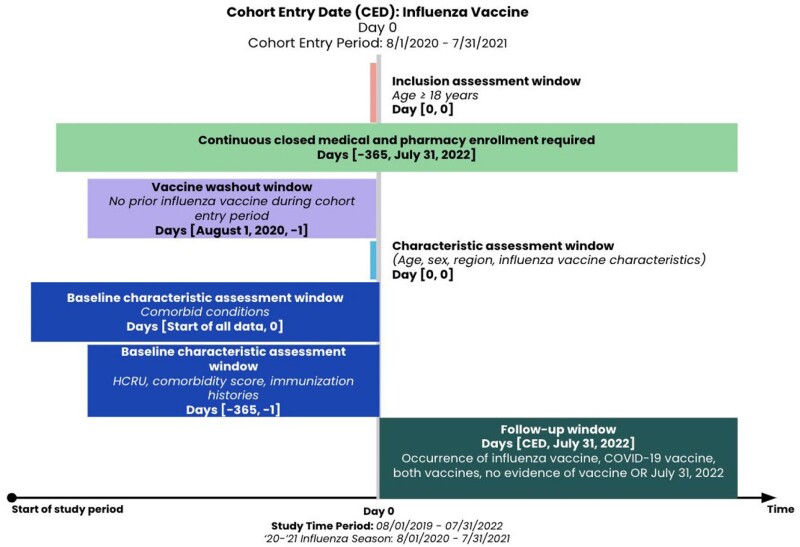
Study Schema

**Figure 2.**
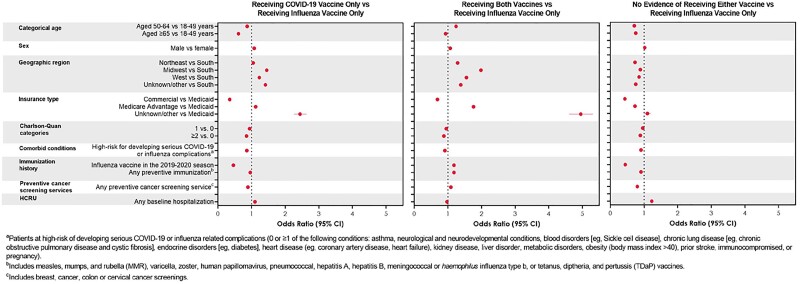
Multivariable Odds Ratios for Factors Associated With Observed Vaccine Pattern in Follow-up

**Methods:**

A cohort of adults ≥18 years with evidence of an influenza vaccination administered between August 1, 2020, and July 31, 2021 (index date) was generated using open and closed administrative claims sourced from HealthVerity. Fig 1 includes additional inclusion and exclusion criteria, subject characteristics, and follow-up windows. Baseline characteristics and time-to-subsequent vaccine administration after index date were assessed via descriptive statistics. Multinomial logistic regression was used to assess the association between subject characteristics and receipt of subsequent influenza and COVID-19 vaccines.

**Results:**

Of 3,043,095 influenza vaccinated adults, 13.7% had a subsequent influenza vaccination, 21.1% had a subsequent COVID-19 vaccination, and 50.7% had both through July 31, 2022. Following the index date, the median (IQR) time to a subsequent influenza vaccine was 373 (353, 396) days and 185 (144, 383) days for a subsequent COVID-19 vaccine. Among those who received both vaccines, most (64.1%) received a COVID-19 vaccine followed by influenza. Prior influenza vaccine was associated with subsequent receipt of both vaccines vs influenza only (OR, 95% CI: 1.18, 1.18-1.19). Adults ≥65 years and those considered high-risk for serious complications were slightly less likely to receive both vaccines compared to influenza only (Fig 2; OR, 95% CI, ≥65 years: 0.93, 0.92-0.94; high-risk: 0.91, 0.90-0.92). Outcome misclassification could be driving these results.

**Conclusion:**

During influenza seasons impacted by the COVID-19 pandemic, evidence of prior preventive services and immunizations were associated with subsequent influenza and COVID-19 vaccine administration. As the pandemic subsides, there will be a continued need to assess COVID-19 and influenza vaccination patterns to inform future vaccination campaigns, including combination influenza and COVID-19 vaccines.

**Disclosures:**

**Christopher Bush, MPH**, Aetion, Inc.: Employee|Aetion, Inc.: Stocks/Bonds **Katherine E. Mues, PhD**, Aetion, Inc.: Employee **Shaina Desai, MPH**, Aetion, Inc.: Employee **Brent Arakaki, BS**, Aetion, Inc.: Employee **Priyadarshani Dharia, PhD, MD, MPH**, Moderna, Inc.: Salary|Moderna, Inc.: Stocks/Bonds **Parinaz Ghaswalla, PhD**, Moderna, Inc: Employee|Moderna, Inc: Stocks/Bonds **Yoonyoung Park, ScD**, Moderna, Inc.: Employee|Moderna, Inc.: Stocks/Bonds

